# Totally laparoscopic surgery for a hydrocele of the canal of Nuck extending from the abdominal cavity to the subcutaneous space: a case report

**DOI:** 10.1186/s40792-024-01825-w

**Published:** 2024-02-02

**Authors:** Kenichi Nakamura, Takahiko Higashiguchi, Yuko Chikaishi, Kazuhiro Matsuo, Tomoyoshi Endo, Koji Morohara, Kenji Kikuchi, Susumu Shibasaki, Hidetoshi Katsuno, Ichiro Uyama, Koichi Suda, Zenichi Morise

**Affiliations:** 1 Department of Surgery, Fujita Health University Okazaki Medical Center, 1 Azakotanda, Harisaki, Okazaki, Aichi 444-0827 Japan; 2 Department of Surgery, Fujita Health University, 1-98 Dengakugakubo, Kutsukake, Toyoake, Aichi 470-1192 Japan; 3 Advanced Robotic and Endoscopic Surgery, Fujita Health University, 1-98 Dengakugakubo, Kutsukake, Toyoake, Aichi 470-1192 Japan; 4 Collaborative Laboratory for Research and Development in Advanced Surgical Technology, Fujita Health University, 1-98 Dengakugakubo, Kutsukake, Toyoake, Aichi 470-1192 Japan; 5 Collaborative Laboratory for Research and Development in Advanced Surgical Intelligence, Fujita Health University, 1-98 Dengakugakubo, Kutsukake, Toyoake, Aichi 470-1192 Japan

**Keywords:** The canal of Nuck, Hernia repair, Inguinal hernia, Laparoscopic surgery

## Abstract

**Background:**

Hydrocele of the canal of Nuck (HCN) is a rare disease, and its indications for laparoscopic surgery are not well-established.

**Case presentation:**

A 53-year-old woman was referred to our hospital due to an uncomfortable thumb-sized inguinal mass. Preoperative computed tomography scan and magnetic resonance imaging revealed a hydrocele extending from the abdominal cavity around the left deep inguinal ring via the inguinal canal to the subcutaneous space. The patient was diagnosed with HCN protruding into the abdominal cavity and extending to the subcutaneous space. Laparoscopy can easily access the hydrocele protruding into the abdominal cavity. Furthermore, laparoscopic hernioplasty can be superior to the anterior approach for females. Hence, laparoscopic surgery was performed. After transecting the round ligament of the uterus, a tense 3-cm hydrocele was dissected with it. In order to approach the hydrocele distal to the deep inguinal ring, the transversalis fascia was incised medially to the inferior epigastric vessels. The subcutaneously connected hydrocele was excised from the incision. Then, the enlarged deep inguinal ring was reinforced using a mesh with the laparoscopic transabdominal preperitoneal approach. The patient was discharged 2 days postoperatively. Laparoscopic resection can be more effective for a hydrocele protruding into the abdominal cavity as it facilitates an easy access to the hydrocele. Moreover, laparoscopic resection of a hydrocele extending from the inguinal canal to the subcutaneous space via a transversalis fascia incision can be safer, with low risk of injury to the inferior epigastric vessels. The incised transversalis fascia and the enlarged deep inguinal ring due to the HCN were simultaneously repaired with the laparoscopic transabdominal preperitoneal repair. There are two reports on laparoscopic resection via a transversalis fascia incision for HCNs located between the inguinal canal and the subcutaneous space, which does not require intraperitoneal hydrocelectomy. However, this is the first report on laparoscopic resection of large HCNs protruding into the abdominal cavity and extending beyond the inguinal canal into the subcutaneous space via intraperitoneal hydrocelectomy and a transversalis fascia incision.

**Conclusions:**

Laparoscopic surgery with transversalis fascia incision can be useful for HCNs extending from the abdominal cavity to the subcutaneous space.

**Supplementary Information:**

The online version contains supplementary material available at 10.1186/s40792-024-01825-w.

## Background

Hydrocele of the canal of Nuck (HCN) is a rare disease in adult woman [1–3]. HCN is caused by failed obliteration of the distal portion of the canal, which forms a fluid-containing sac. It occurs anywhere along the round ligament of the uterus (RLU), from the deep inguinal ring to the labia [1, 2, 4–6]. The surgical treatments for HCNs include excision of the hydrocele and simultaneous repair of the inguinal defect [7]. Further, surgeries are usually performed using the anterior approach [8]. In recent years, there have been reports on the usefulness of laparoscopic surgery for HCN. However, it is still challenging to safely remove a large hydrocele that extends from the abdominal cavity to the subcutaneous space via laparoscopy alone [9]. Herein, we present a case in which an HCN protruding into the abdominal cavity and extending into the subcutaneous space was successfully managed with totally laparoscopic excision via an incision in the transversalis fascia and laparoscopic hernioplasty.

## Case presentation

**Fig. 1:**
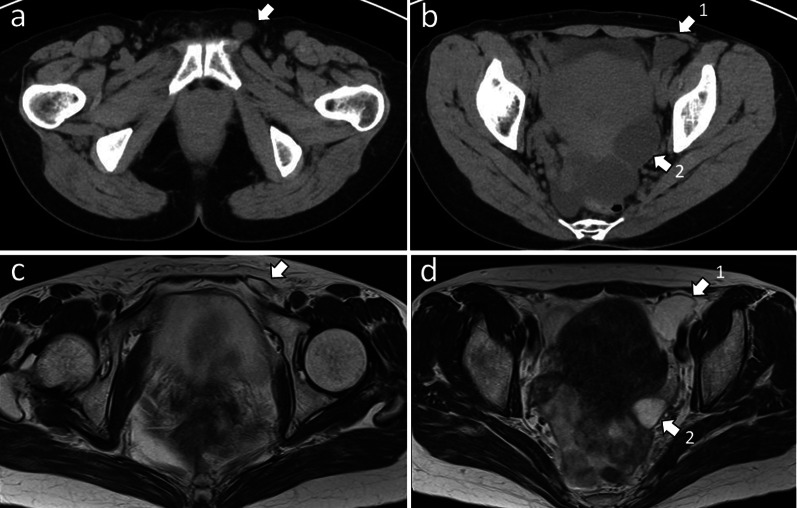
Computed tomography scan and magnetic resonance imaging findings of a hydrocele of the canal of Nuck extending from the abdominal cavity to the subcutaneous. **a** Computed tomography scan findings. Arrow: subcutaneous hydrocele. **b** Computed tomography scan findings. Arrow 1: hydrocele protruding into the abdominal cavity. Arrow 2: ovarian cyst. c. T2-weighted magnetic resonance imaging finding. Arrow: hydrocele in the inguinal canal. d. T2-weighted magnetic resonance imaging finding. Arrow 1: hydrocele protruding into the abdominal cavity. Arrow 2: ovarian cyst

A 53-year-old woman was referred to our hospital due to a thumb-sized mass and discomfort in the left inguinal region. Computed tomography (CT) scan and magnetic resonance imaging (MRI) showed a hydrocele in the left inguinal region, which spread from the abdominal cavity via the inguinal canal to the subcutaneous space, and an ovarian cyst on the dorsal surface of the uterus (Fig. 1a–d). Thus, the patient was preoperatively diagnosed with HCN and ovarian cyst. A totally laparoscopic approach was planned due to the following: first, the previous reports have shown that resection from a transversalis fascia incision for HCN from the inguinal canal to the subcutaneous space can be safely performed [8, 10]. Second, large hydroceles protruding into the abdominal cavity can be resected laparoscopically more easily than the anterior approach. Third, the transversalis fascia incision and the enlarged deep inguinal ring due to the HCN can simultaneously be repaired via transabdominal preperitoneal (TAPP) repair.

**Fig. 2:**
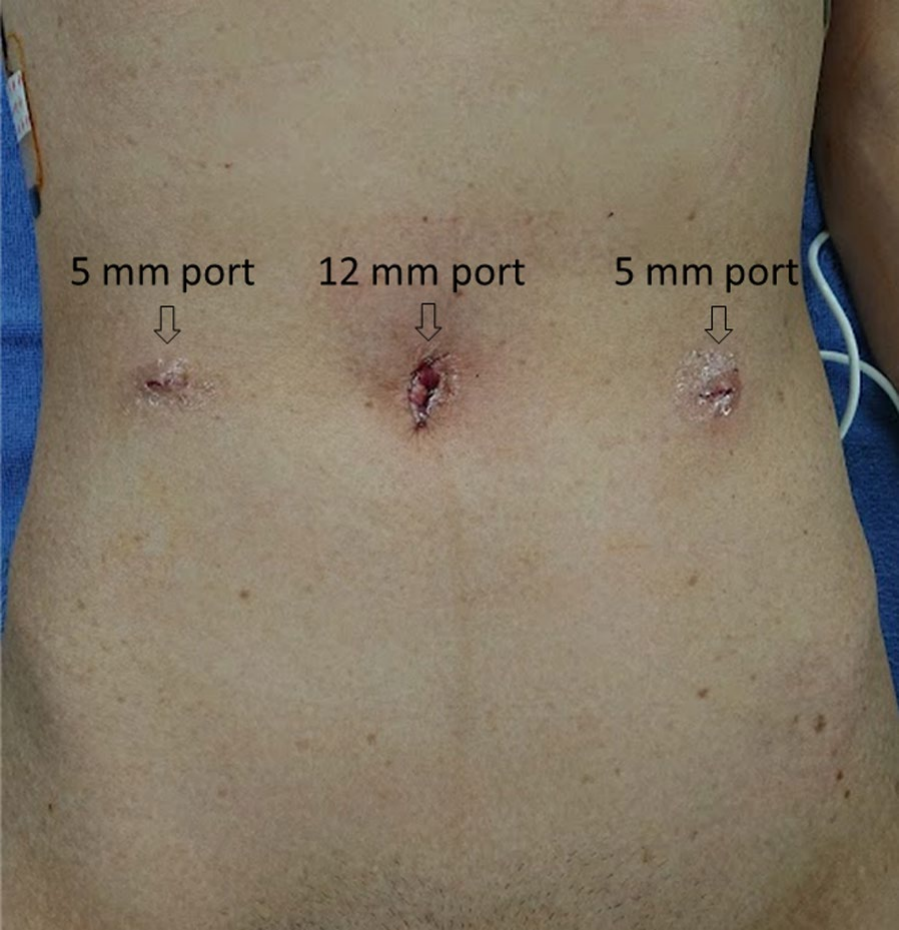
Port placement

**Fig. 3:**
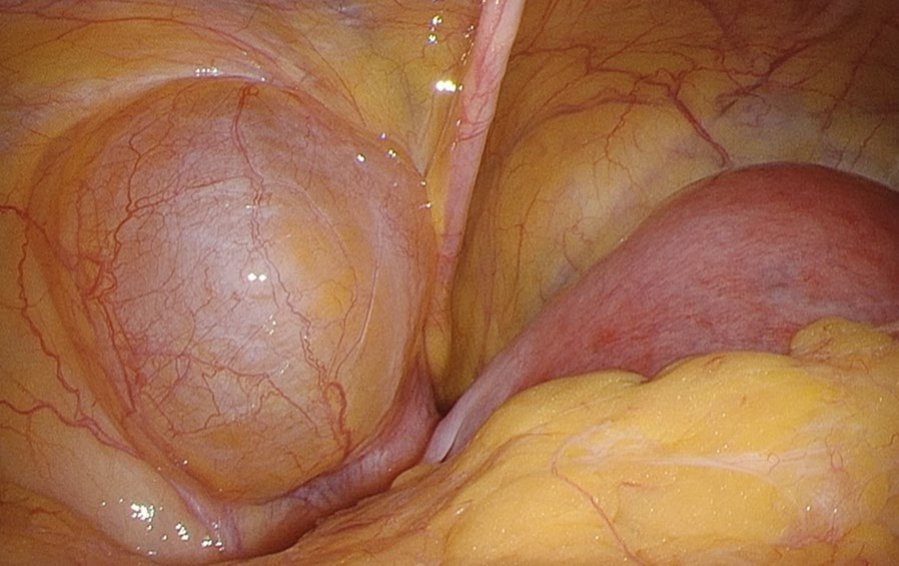
Intraoperative findings of hydrocele of the canal of Nuck protruding into the abdominal cavity

**Fig. 4:**
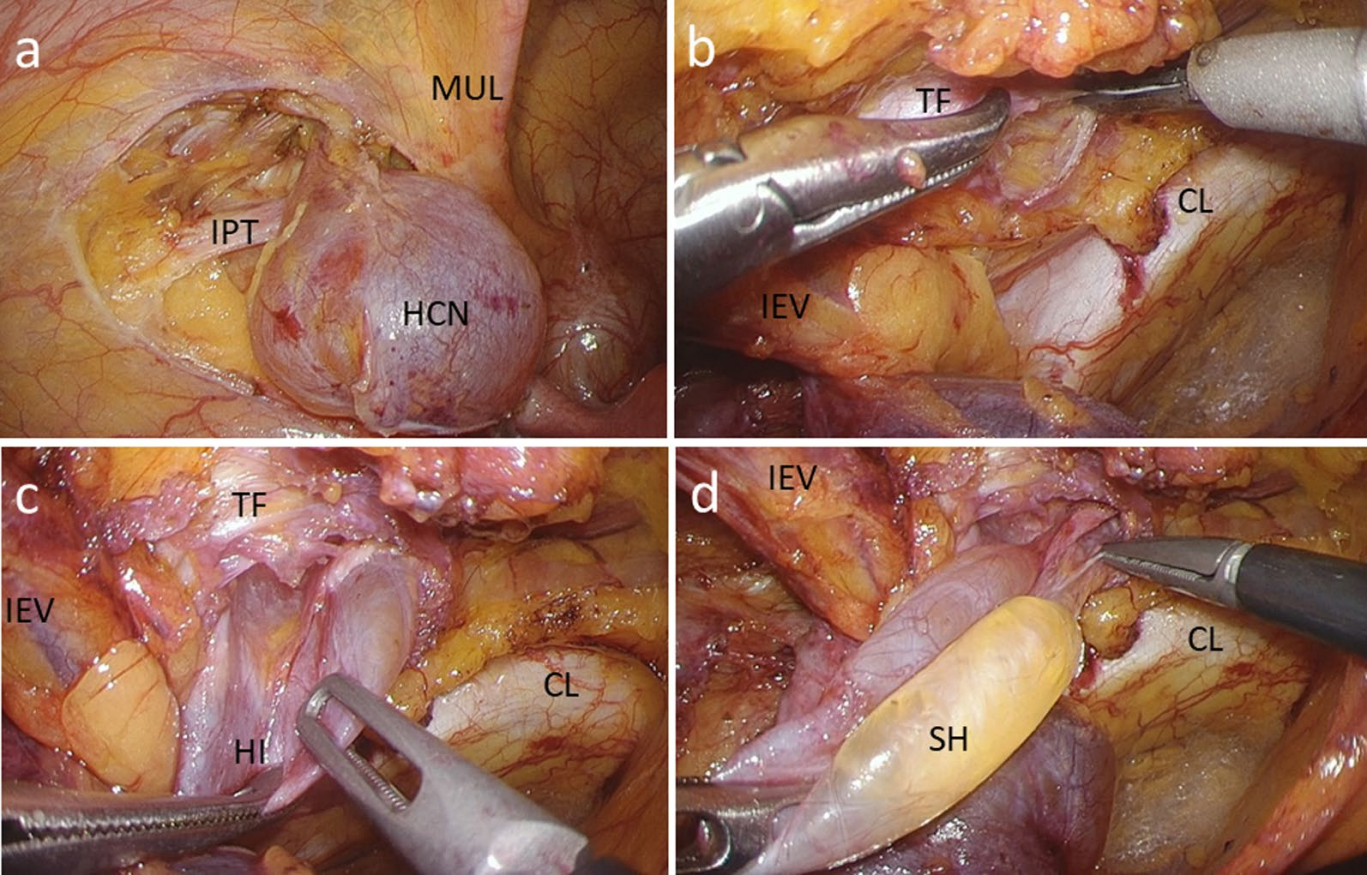
Intraoperative findings during resection of a hydrocele of the canal of Nuck. **a** Resection of the hydrocele protruding into the abdominal cavity. **b** Incision of the dorsal transversalis fascia. **c** Dissection of the hydrocele in the inguinal canal via a transversalis fascia incision. **d** Dissection of the subcutaneous hydrocele from the transversalis fascia incision. IPT, iliopubic tract; HCN, hydrocele of the canal of Nuck**;** MIL, medial umbilical ligament; IEV, inferior epigastric vessel; TF, transversalis fascia; CL, Cooper’s ligament; HI, hydrocele in the inguinal canal; SH, subcutaneous hydrocele

**Fig. 5:**
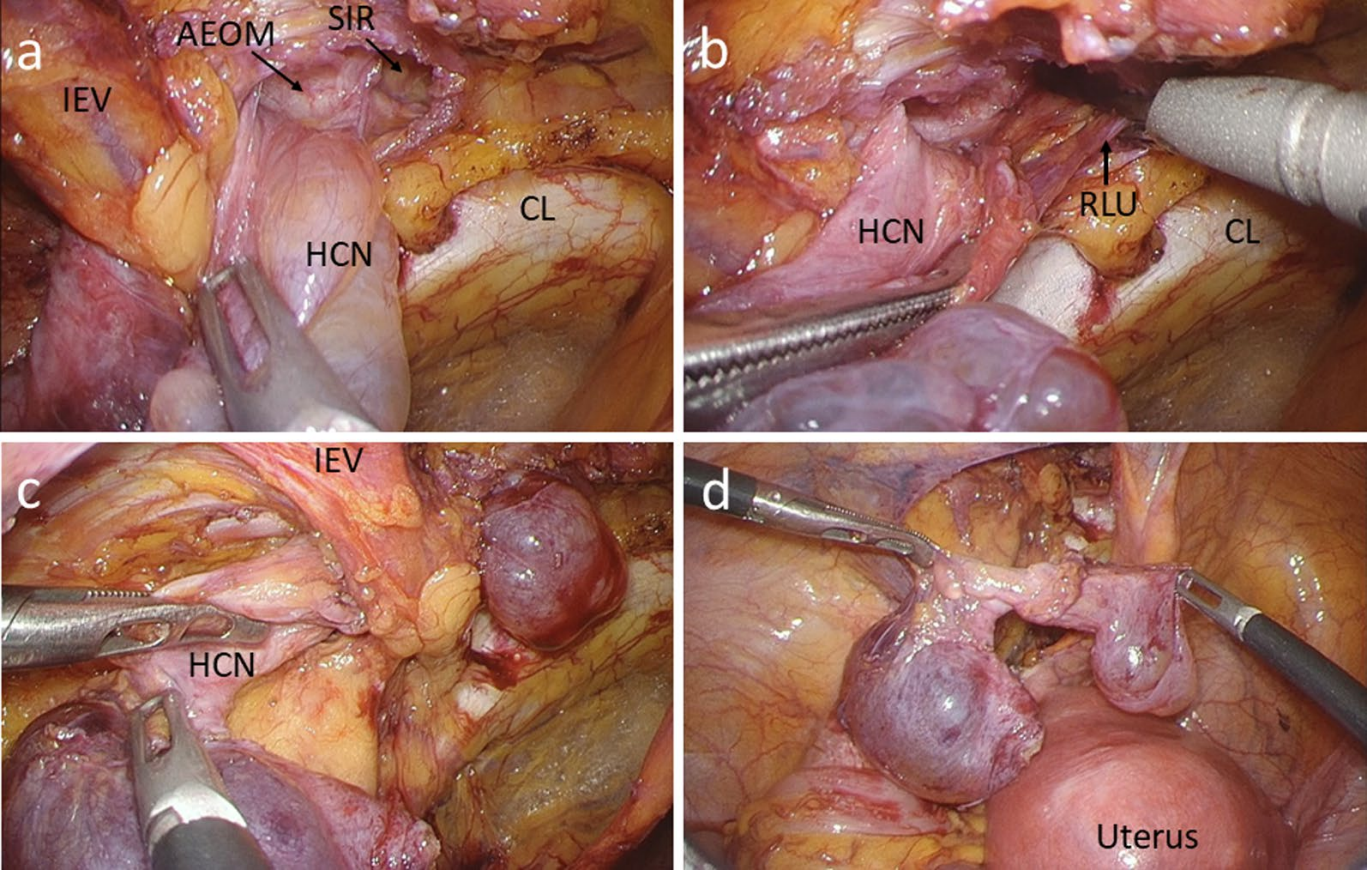
Intraoperative findings during resection of a hydrocele of the canal of Nuck. **a** Complete excision of the subcutaneous hydrocele. No remnant hydrocele could be observed subcutaneously via the superficial inguinal ring. **b** Distal round ligament of the uterus transection. **c** The hydrocele passed in front of the inferior epigastric vessels and extracted from the deep inguinal ring. d. Hydrocele excised without rupture. AEOM, aponeurosis of external oblique muscle; SIR, superficial inguinal ring; RLU, round ligament of the uterus

**Fig. 6:**
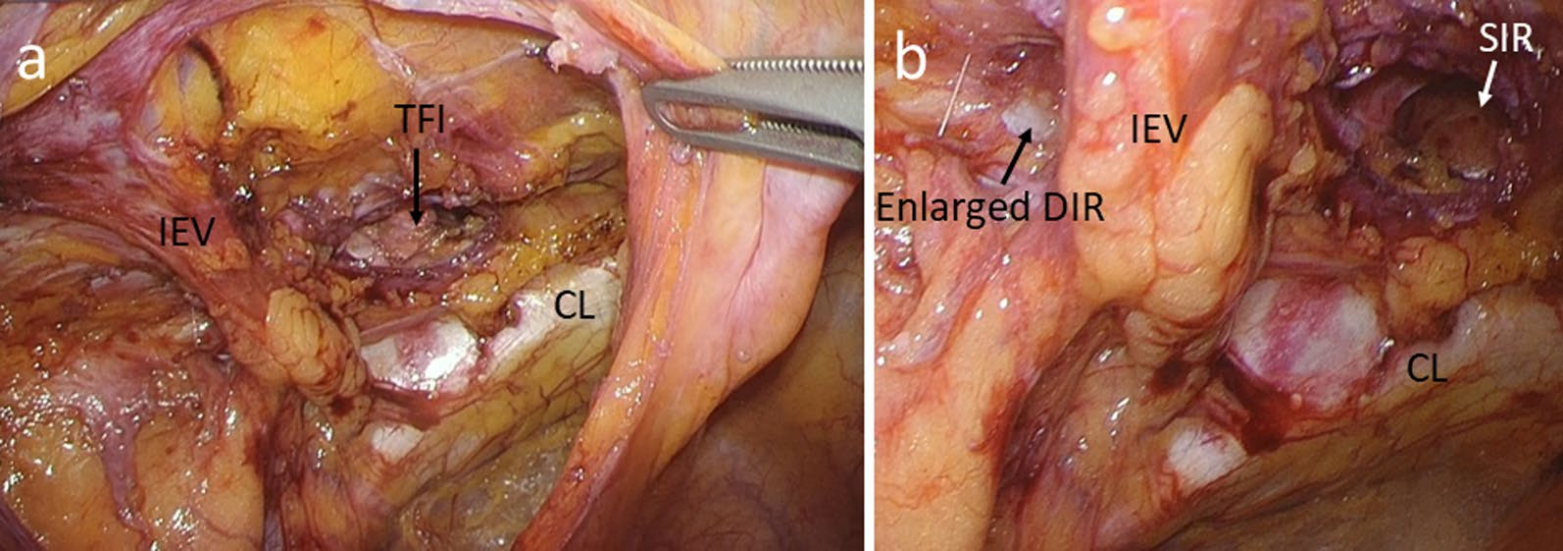
Myopectineal orifice before mesh repair. **a** Myopectineal orifice after hydrocele resection. **b** Enlargement of the deep inguinal ring and defects in the posterior wall of the inguinal canal. The superficial inguinal ring was observed via the transversalis fascia incision. TFI, transversalis fascia incision; DIR, deep inguinal ring

**Fig. 7:**
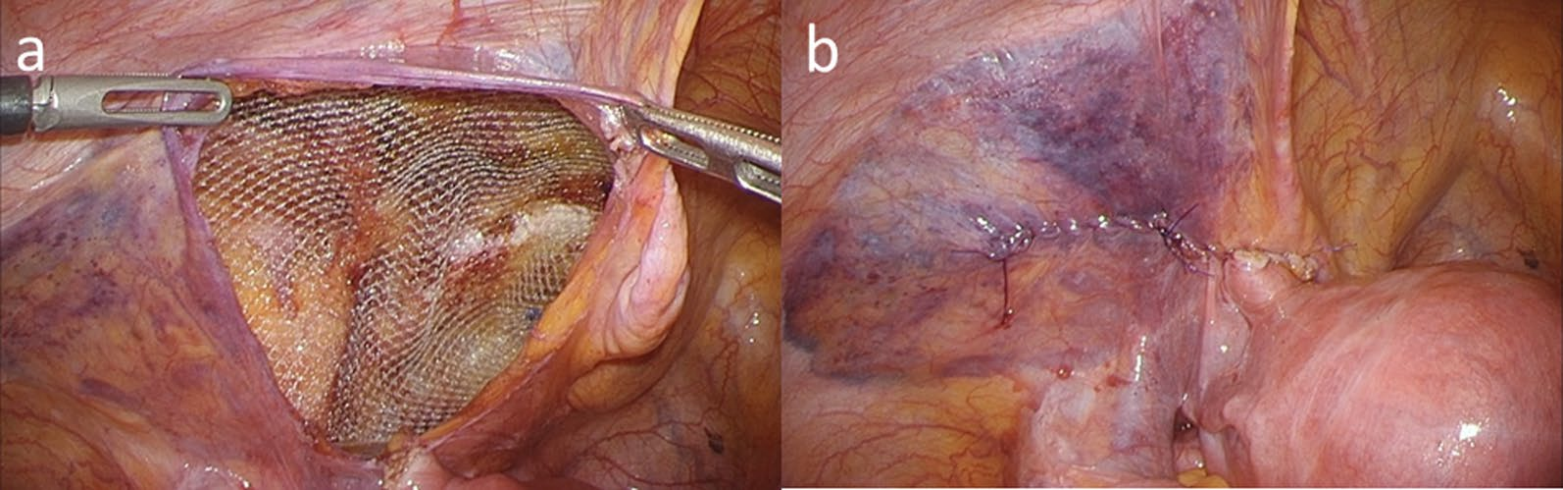
Repair of the myopectineal orifice using a mesh (transabdominal preperitoneal repair). **a** Mesh repair and mesh fixation with an absorbent tacker. **b** Peritoneal closure

We used three ports for the laparoscopic approach (Fig. 2). A 12-mm port was inserted at the umbilicus, and laparoscopic examination revealed a tense 3-cm hydrocele protruding into the abdominal cavity at the left deep inguinal ring (Fig. 3). In addition, ovarian and endometrial cysts were found on the left ovary. A 5-mm port was inserted in the right and left lateral abdomen. First, a gynecologist performed an intra-abdominal exploration for endometriosis and incisional ablation of an endometriotic cyst. He found that the hydrocele was not related to endometriosis. Subsequently, laparoscopic HCN resection was performed. After transecting the RLU, the hydrocele was dissected along with the RLU (Fig. 4a). To approach the distal part of the hydrocele from the deep inguinal ring, the dorsal transversalis fascia was incised medially to the inferior epigastric vessels (Fig. 4b). Moreover, the subcutaneous and intrainguinal canal part of the hydrocele was continuously dissected from the incision (Figs. 4c, d, and 5a). The RLU was transected as distally as possible (Fig. 5b). The distal part of the hydrocele with RLU was extracted into the abdominal cavity via the deep inguinal ring passing beside the inferior epigastric vessels without rupturing (Fig. 5c, d). Specimens were placed in a plastic bag and retrieved via the umbilicus. Then, the enlarged deep inguinal ring was covered using a mesh with the laparoscopic TAPP approach (Figs. 6a, b, 7a, and b, Additional file 1: video). The operative time was 114 min, and the total volume of blood loss was 2 mL. The postoperative course was uneventful, and the patient was discharged on postoperative day 2. No signs of hydroceles, inguinal pain or inguinal hernia were detected 3 months after the surgery. Based on the pathological finding, the hydrocele was a benign cyst, and there were no findings of endometriosis.

## Discussion

The canal of Nuck is the female equivalent of the male processus vaginalis, a perineal protrusion that extends through the inguinal canal and ends at the labia majora [11]. This canal commonly obliterates early in life. However, in some cases, it fails to partially or completely close, which is likely to lead to hydrocele or hernia of the pelvic contents. In planning a surgical strategy, a preoperative diagnosis of the HCN location is required not only in physical examination but also in ultrasonography, CT scan, or MRI [7]. Recently, there have been reports showing the usefulness of laparoscopic examination as part of the treatment strategy [10, 11]. Wang et al. classified HCN into the following categories according to the anatomical position for a better intraoperative understanding: type A, HCN is located in the subcutaneous space over the inguinal canal; type B, HCN is located in the inguinal canal; type C, HCN is limited to the deep inguinal ring; and type D, HCN spreads from the deep inguinal ring to the inguinal canal or subcutaneous space [4]. After the localization is clarified, it is necessary to decide whether the laparoscopic or anterior approach with or without a mesh should be used. In recent years, there have been a few reports on successful totally laparoscopic resection and hernioplasty for HCN. To the best of our knowledge, there are only 10 reports (*n* = 13) including our case [4, 5, 8, 10, 12–16] (Table 1). According to the HCN location, Wang et al. recommended the following approaches: anterior approach for type A, laparoscopic TAPP exploration plus the anterior approach for type B, laparoscopic TAPP approach for type C, and laparoscopic TAPP or totally extraperitoneal approach plus the anterior approach for type D. In the present case, preoperative physical examination, CT scan, and MRI were conducted to diagnose type D hydrocele accompanied with a large protrusion into the abdominal cavity and enlargement of the deep inguinal ring. Subsequently, totally laparoscopic hydrocele resection with transversalis fascia incision and laparoscopic hernioplasty using a mesh were performed. The procedure was then successfully completed.

**Table 1:** Reports on successful totally laparoscopic resection and hernioplasty for hydrocele of the canal of Nuck

No	Authors	Year	Hernia characteristics				Procedures		
			HCN Classification by Wang (type)	Size (mm)	Protrusion into the abdominal cavity	Enlargement of the DIR	Transversalis fascia incision	Dissection from the DIR	Hernioplasty
1	Matsumoto T	2014	D	45	+	+	−	+	TEP
2	Qureshi NJ	2014	B	40 × 30	−	+	−	+	TAPP
3	Chihara N	2020	A (or A + B)	30 × 28	−	+	+	−	TAPP
4	Kojima S	2020	B	ND (thumb size)	−	−	+	−	TEP
5	Shahid F	2020	D	84 × 26	+	+	−	+	TAPP
6	Fikatas P	2020	UI	30 × 20	ND	ND	−	+	TAPP
7	Fikatas P	2020	D	ND	+	ND	−	+	TAPP
8	Fikatas P	2020	UI	30 × 40	ND	ND	−	+	TAPP
9	Fikatas P	2020	D	ND	ND	+	−	+	TAPP
10	Wang L	2021	B	40 × 30	−	−	−	+	TAPP
11	Tominaga M	2022	D	50 × 10	+	+	−	+	TAPP
12	Almagushi N	2023	B	ND	−	+	−	+	TAPP
13	Our case	2023	D	70 × 30	+	+	+	−	TAPP

*HCN* hydrocele of the canal of Nuck, *DIR* deep inguinal ring, *UI* unidentifiable, *ND* not described, *TAPP* transabdominal preperitoneal approach, *TEP* totally extraperitoneal approach

In the present case, the laparoscopic approach was considered better for two reasons. First, the large hydrocele protruding into the abdominal cavity was easily accessible. Second, the laparoscopic approach may be superior to the anterior approach in inguinal hernioplasty using a mesh particularly in women.

Regarding accessibility to hydroceles, in 5 of the abovementioned 13 cases, including ours, the patients underwent totally laparoscopic resection for HCN protruding into the abdominal cavity, except in three cases in which the detail was not provided. In such cases, laparoscopic approach may allow the direct visual resection of the protruding hydrocele in a larger operative field compared with the anterior approach, which enlarges the deep inguinal ring and is proceeded with deep dissection.

**Fig. 8:**
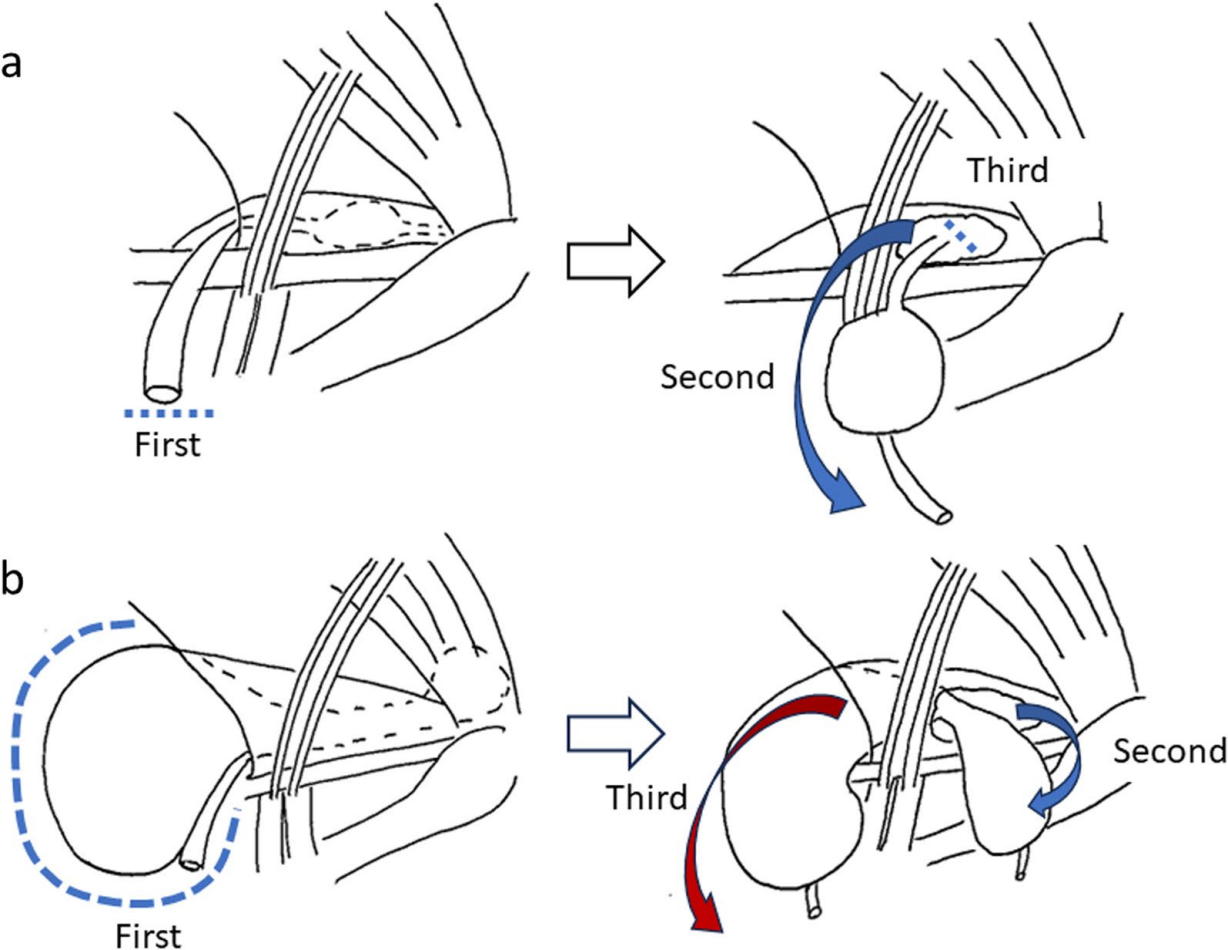
Schema of hydrocelectomy with the transversalis fascia incision. **a** Resection of a hydrocele located in the inguinal canal to the subcutaneous space. First, the round ligament of the uterus was transected. Second, the hydrocele with the ligament of the uterus was excised via transversalis fasciotomy. Third, the distal part of the round ligament of the uterus was transected. **b** Resection of the hydrocele extending from the abdominal cavity to the subcutaneous space (current procedure). First, after transection of the round ligament of the uterus, the hydrocele protruding into the abdominal cavity was resected. Second, the subcutaneous and intrainguinal canal part of the hydrocele was dissected from the transversalis fascia incision. Then, the distal part of the round ligament of the uterus was transected. Third, the distal part of the hydrocele was extracted into the abdominal cavity via the deep inguinal ring passing beside the inferior epigastric vessels

Regarding accessibility to distal hydroceles, in 3 of the abovementioned 13 cases, including ours, the HCN was laparoscopically excised via the transversalis fascia incision. In two cases except ours, the HCNs were located only in the inguinal canal to the subcutaneous space that does not require an intraperitoneal hydrocelectomy (Fig. 8a). To the best of our knowledge, this is the first report on laparoscopic resection of large HCNs protruding into the abdominal cavity and extending beyond the inguinal canal into the subcutaneous space with transversalis fascia incision (Fig. 8b). Compared with the abovementioned two cases, our case involved a wider extent of hydrocele and increased procedural difficulty. However, the hydrocele could be safely resected (Fig. 8a, b). In the remaining 10 cases, the hydrocele was dissected via the deep inguinal ring and was excised. With our method, the end of the hydrocele could be reached easier, and visualization could be better. Moreover, the risk of damage to the inferior epigastric vessels was lower than that of the procedure via the deep inguinal ring [8]. Wang et al. revealed that if the inguinal canal is extremely deep or the HCN is significantly large, it is challenging to successfully free the distal end of the HCN via the deep inguinal ring on laparoscopy [4]. However, transversalis fascia incision requires repair of the posterior wall of the inguinal canal with or without a mesh. In the present case, the deep inguinal ring was enlarged due to the hydrocele, and the myopectineal orifice finally required repair using a mesh. Therefore, no additional repairing procedure is needed for the transversalis fascia incision.

Regarding hernioplasty using a mesh, laparoscopic TAPP repair was performed. Laparoscopic hernioplasty is more advantageous in terms of cosmetic aspects. Moreover, it allows for an earlier return-to-normal activity or work and causes less chronic pain compared with the anterior approach (Lichtenstein technique) [17]. In addition, the European Hernia Society guidelines optionally recommend laparoscopic hernioplasty for female with inguinal hernias, which can cover both the inguinal and femoral orifices simultaneously, to prevent femoral recurrence, which are more frequent in females [17]. In the abovementioned reports on totally laparoscopic surgery, all patients had hernioplasty using a mesh. However, two of them had no enlargement of deep inguinal ring without the need for mesh repair [4, 8]. Wang et al. revealed that if the deep inguinal ring is not enlarged after laparoscopic observation, hydrocele resection using the anterior approach alone can be required without mesh repair. The use of a mesh should be reconsidered from the standpoint of cost and time [4]. Therefore, in such cases, totally laparoscopic surgery may be indicated if cosmetic advantages exceed the disadvantages of mesh use.

Hence, our totally laparoscopic procedure has several advantages. However, it also has several disadvantages. That is, if the subcutaneous hydrocele is extremely large, excision of the subcutaneous hydrocele from the superficial inguinal ring can be more challenging. Therefore, if complete resection of the hydrocele is difficult due to a large subcutaneous hydrocele, conversion from the laparoscopic procedure to the anterior approach should be considered.

Totally laparoscopy with transversalis fascia incision performed in the present report may be reproducible and applicable to all types of HCN. However, for hydrocele, only located between the inguinal canal and the subcutaneous space and without deep inguinal ring enlargement, the anterior approach can be used for hydrocelectomy without mesh hernioplasty.

Thus, an HCN protruding into the abdominal cavity, with an enlarged deep inguinal ring leading to the distal portion of the inguinal canal, is a suitable indication for totally laparoscopic surgery with transversalis fascia incision.

## Conclusions

In the present case, totally laparoscopic HCN resection via the transversalis fascia incision and laparoscopic TAPP repair were useful for large HCNs that protrude into the abdominal cavity and that are continuous with the subcutaneous space.

### Supplementary Information

**Additional file 1. **Operative video of the totally laparoscopic surgery for a hydrocele of the canal of Nuck extending from the abdominal cavity to the subcutaneous space.

## Data Availability

Not applicable.

## Abbreviations

HCN: Hydrocele of the canal of Nuck
RLU: Round ligament of the uterus
CT: Computed tomography
MRI: Magnetic resonance imaging
TAPP: Transabdominal preperitoneal
